# A Low Power Digital Accumulation Technique for Digital-Domain CMOS TDI Image Sensor

**DOI:** 10.3390/s16101572

**Published:** 2016-09-23

**Authors:** Changwei Yu, Kaiming Nie, Jiangtao Xu, Jing Gao

**Affiliations:** School of Electronic Information Engineering, Tianjin University, 92 Weijin Road, Nankai District, Tianjin 300072, China; yuchangwei@tju.edu.cn (C.Y.); niekaiming@tju.edu.cn (K.N.); xujiangtao@tju.edu.cn (J.X.)

**Keywords:** CMOS TDI image sensor, accumulation technique, coarse quantization, fine quantization

## Abstract

In this paper, an accumulation technique suitable for digital domain CMOS time delay integration (TDI) image sensors is proposed to reduce power consumption without degrading the rate of imaging. In terms of the slight variations of quantization codes among different pixel exposures towards the same object, the pixel array is divided into two groups: one is for coarse quantization of high bits only, and the other one is for fine quantization of low bits. Then, the complete quantization codes are composed of both results from the coarse-and-fine quantization. The equivalent operation comparably reduces the total required bit numbers of the quantization. In the 0.18 µm CMOS process, two versions of 16-stage digital domain CMOS TDI image sensor chains based on a 10-bit successive approximate register (SAR) analog-to-digital converter (ADC), with and without the proposed technique, are designed. The simulation results show that the average power consumption of slices of the two versions are $6.47\times 10^{-8}$ J/line and $7.4\times 10^{-8}$ J/line, respectively. Meanwhile, the linearity of the two versions are 99.74% and 99.99%, respectively.

## 1. Introduction

Linear image sensors capture images by scanning the target scene. In low light or high speed scanning condition, the integration time of each pixel is too short to collect enough photons, which results in low signal-to-noise ratio (SNR). The time delay integration (TDI) images’ sensor technology adopts more rows of pixels to scan the same scene, and the integrated signals are accumulated together, which extends the integration time equivalently. Thus, a better SNR can be achieved using a TDI image sensor. Compared with the regular linear image sensor, the accumulated signal and uncorrelated noise of a TDI image sensor are amplified by a factor *N* and square root of *N* respectively, which results in a square root of *N* times improvement of SNR, where *N* is the stage of accumulation. TDI image sensors are suitable in the situations where a line-scan system is required for high quality and low noise imaging even under low illuminations and high speed scanning [1,2]. The TDI technology could be easily implemented in a charge coupled device (CCD) [3] because it allows noiseless accumulation of signals in the charge domain. However, the CCD requires high supply voltage, which results in large power consumption. Additionally, due to the incompatibility with the CMOS process, the CCD could not integrate with other functions on the same chip with the sensor [4]. In recent years, TDI image sensors implemented with the CMOS process have been reported [5,6,7,8,9,10,11,12,13,14]. Compared with the CCD, CMOS TDI image sensors could successfully integrate various analog and digital circuits as an on-chip camera [4]. Such integration further reduces the power consumption and size [4].

There are typically three kinds of accumulation methods: charge-domain accumulation scheme [5,6,7,8], analog-domain accumulation scheme [9,10,11] and digital domain accumulation scheme [12,13,14] for CMOS TDI image sensors. In the charge-domain accumulation scheme, the noiseless accumulation enables good SNR. However, the charge transfer efficiency and full well capacity, which strongly depend on process, limit its momentum of development [6,7]. With respect to the analog-domain accumulation scheme, the generated signals from pixels are firstly accumulated by the analog accumulator, and then the accumulated result is quantized by column analog-to-digital converter (ADC) [9,10]. Because the maximum accumulated voltage depends on the supply voltage, the dynamic range of the analog-domain CMOS TDI image sensor is limited by the supply voltage [9,10]. Since the analog memory cell of the analog accumulator is implemented with capacitors, the accumulator area is considerably large [9,10]. In the digital domain accumulation scheme, the outputs of pixels are firstly quantized by column ADC, and then the corresponding digital results are added together by the digital accumulator [14]. The memory cell of the digital accumulator is implemented by digital registers, so the digital accumulator area is less than an analog accumulator with the same accumulation stages [14]. In order to make the most use of the quantization range of ADC, the output signals of pixels are usually amplified several times before being sent to the ADC, which results in better SNR than that of analog-domain accumulation [13,14]. However, at high speed scanning and high accumulation stages, a large amount of data generated by ADC bring in more power consumption. In this paper, a low power digital accumulation technique for a CMOS TDI image sensor is proposed to reduce power consumption without degrading the rate of imaging by means of downscaling the bit numbers of the quantization.

The remainder of this paper is organized as follows. In Section 2, we firstly describe the principle of the low power accumulation technique, the optimized implementation scheme is then discussed, and finally a behavioral simulation is completed with MATLAB (R2011b, MathWorks, Natick, MA, USA). Section 3 presents the circuit implementation scheme of the low power accumulation technique and the simulation results. A brief conclusion is drawn in Section 4.

## 2. Low Power Digital Accumulation Technique

### 2.1. Principle of the Low Power Digital Accumulation Technique

**Figure 1:**
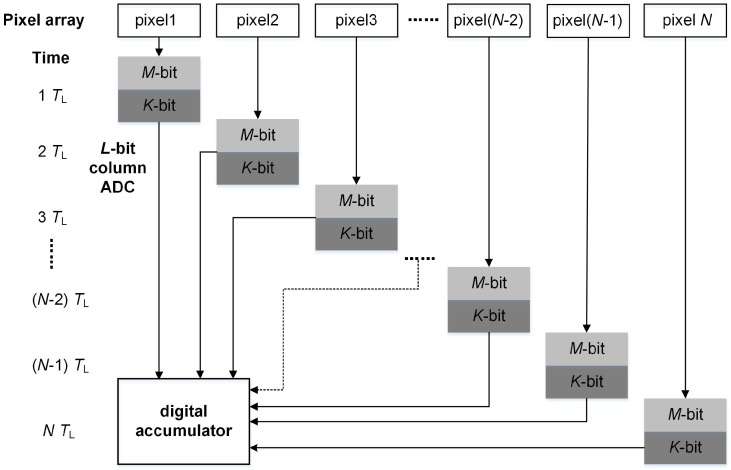
Traditional digital domain accumulation scheme.

The accumulation scheme of the traditional *N*-stage digital domain CMOS TDI image sensor with *L*-bit column ADC, where L=(M+K) is shown in Figure 1. During the exposure time, the exposed object moves at a constant speed relative to the pixel array. The line time ($T_{L}$) is defined as the time that the exposed object moves over exactly one pixel pitch, and the line rate is defined as 1/$T_{L}$. The operation procedure is briefly described as follows. At the first $T_{L}$, the object is captured by pixel1. At the end of the integration of pixel1, the exposure result is sent to the column ADC and quantized into *L*-bit digital data. Then, the data is sent to the digital accumulator. At the second $T_{L}$, the same object is captured by pixel2 and its quantized data is also sent to the digital accumulator. In the same way, the object is exposed *N* times, and the corresponding quantized data are added in the digital accumulator. With the imaging flow above, the ADC converts L×N bits data at the *N* accumulation stage.

Ideally, the output results of pixels detecting the same object should be same. However, the noise may bring some variation in the low-bit codes of the quantized data. The influenced bit numbers depend on the SNR of output signals. The high bits of the quantized results remain the same among pixels detecting the same object. Therefore, there is no need to fully quantify every output signal of pixels. A low power digital accumulation technique for CMOS TDI image sensors is proposed by introducing coarse quantization and fine quantization, aiming at less data and lower power consumption. Based on the discussion above, when the output signals of pixels are quantized into *L*-bit codes by ADC, only the low *K* bits varies. The rest of the high bits is indicated by *M* and L=(M+K). The high *M* bits of the quantized results of the same object are the same.

**Figure 2:**
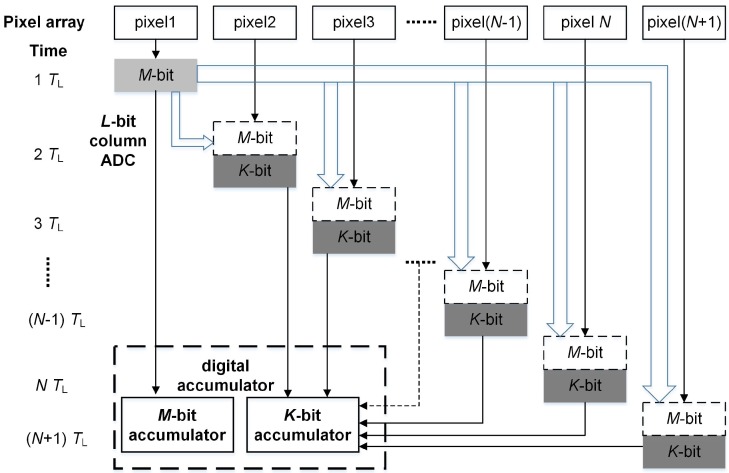
Low power digital domain accumulation scheme.

The proposed low power digital domain accumulation scheme with *L*-bit column ADC is shown in Figure 2. The digital accumulator is divided into high *M*-bit accumulator and low *K*-bit accumulator. At the first $T_{L}$, the object is captured by pixel1. At the end of integration of pixel1, its analog result is sent to the column ADC and only coarsely quantized into high *M* bits, where the low power accumulation scheme differs from the traditional accumulation scheme. Then, the high *M*-bit codes are sent to the high *M*-bit accumulator. During the second $T_{L}$, the same object is captured by pixel2 and its analog result is sent to the column ADC for fine quantization. It only needs to be quantized into low *K* bits, as the high *M* bits have been already quantized in the first $T_{L}$. Then, the low *K*-bit codes are sent to the low *K*-bit accumulator. In the same way, the remaining pixels are all quantized with only low *K* bits and then transmitted to the low *K*-bit accumulator. Finally, the complete quantization result is obtained by combining high *M* bits with low *K* bits. To accomplish *N*-stage accumulation, the ADC needs to convert (M+N×K) bits data in the proposed low power accumulation scheme. Compared with the traditional accumulation scheme, the ADC saves (N-1)×M bits conversion time with the low power accumulation scheme.

In order to increase the quantization precision of the high *M* bits, some lines of pixels are arranged to repeat the coarse quantization, and then the quantized high *M*-bit codes are averaged. Assuming that *P* lines of pixels are used to repeat the coarse quantization, the ADC of the low power digital accumulation scheme will save (N-P)×M bits conversion time compared with the traditional digital accumulation scheme for a complete *N*-stage accumulation. The low power digital domain CMOS TDI image sensor needs much less conversion time at the same conversion speed and accumulation stages, which could achieve lower power consumption than the traditional digital domain CMOS TDI image sensor. According to the operation procedure, successive approximate register (SAR) ADC is very suitable for the low power digital domain accumulation scheme.

### 2.2. Analysis and Optimization of the Coarse Quantization Bit Numbers and Coarse Quantization Times

**Figure 3:**
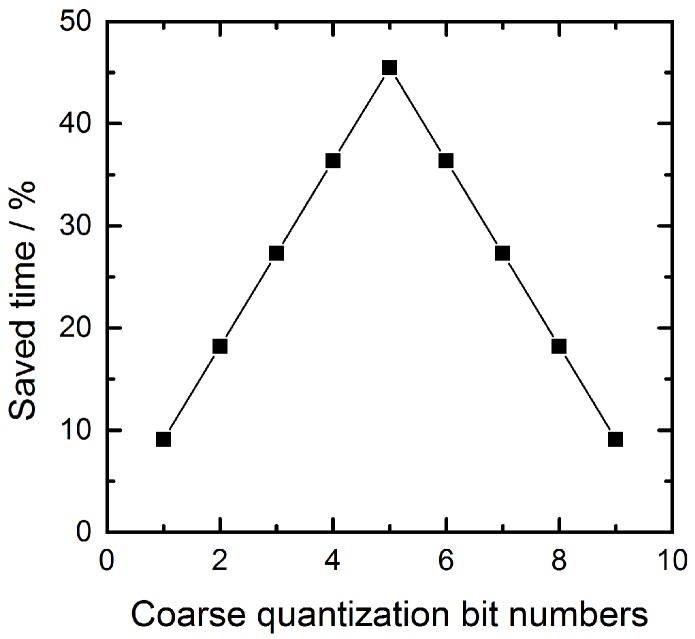
Relation curve between the saved time of SAR ADC and coarse quantization bit numbers.

To analyze and optimize the coarse quantization bit numbers and coarse quantization times of the low power accumulation scheme, a 16-stage digital domain CMOS TDI image sensor based on 10-bit column parallel SAR ADC is established. The total conversion time of a SAR ADC is divided into two parts: sampling time and quantization time. While the sampling time is negligible, it needs 11 clock cycles to complete one conversion for a 10-bit SAR ADC. In the low power accumulation scheme, the full 10-bit conversion is divided into a coarse one and a fine one. Then, the saved time of SAR ADC depends on the maximum consumption time between the coarse quantization and the fine quantization, and the saved time could be roughly calculated as

(1) $$\mathrm{Saved}\;\mathrm{time}=\frac{11-\max(K,\;\;M)-1}{11}\times 100\%,$$

where *M* is the coarse quantization bit numbers and *K* is the fine quantization bit numbers. The relationship between the coarse quantization bit numbers and the saved time of the ADC conversion is illustrated in Figure 3. When the coarse quantization bit numbers are 5-bit, the saved time could reach a maximum value of 45.45%.

**Figure 4:**
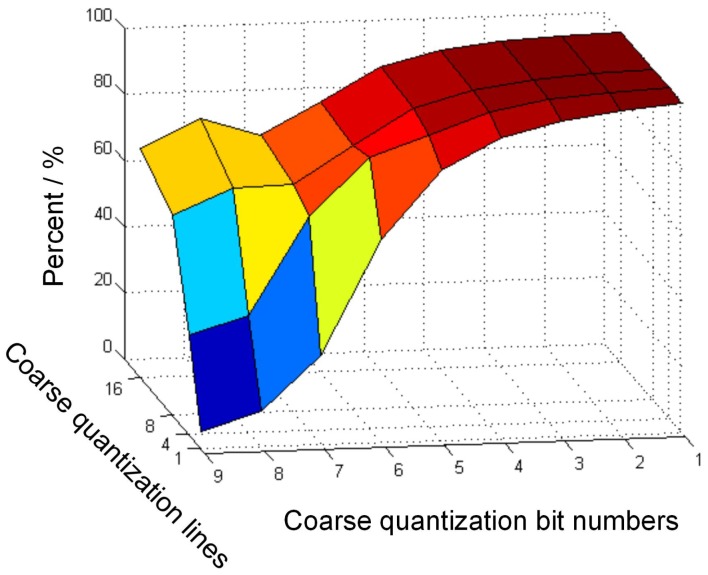
Comparing results between the low power accumulation scheme and the traditional accumulation scheme.

Because the output signals of pixels are influenced by the pixel noise and readout circuit noise, the precision of the coarse quantization decreases with the increase of the coarse quantization bit numbers. Therefore, the precision of the coarse quantization should be taken into account considering the saved time. More than one line of pixels can be used to repeat the coarse quantization procedure to improve the precision. A behavioral simulation is implemented with MATLAB to explore the precision difference between the traditional accumulation scheme and the low power accumulation scheme that adopts different coarse quantization bit numbers and different lines of pixels for coarse quantization. The simulation result is shown in Figure 4. The difference between the results of the proposed scheme and the traditional digital accumulation scheme is indicated by probability that the quantization results of the proposed scheme can reach that of the traditional one. As shown in Figure 4, the probability decreases with the increase of the coarse quantization bit numbers and it is proportional to the lines of pixels for coarse quantization. Considering the quantization precision, chip area and saved time, 4-bit coarse quantization and four-line pixels for coarse quantization are selected for a 16-stage low power digital domain CMOS TDI image sensor with 10-bit SAR ADC. The analysis method is suitable for digital domain CMOS TDI image sensors with various accumulation stages.

As the sampling time of SAR ADC is taken into consideration, a complete conversion needs (11+H) clock periods for a 10-bit SAR ADC in the actual design, where *H* is the number of clocks for sampling. If an *N*-stage low power digital domain CMOS TDI image sensor adopts the above optimized parameters, a complete *N*-stage accumulation consumes (N+4)×(11+H-4) clock periods. With the same accumulation stages, a complete *N*-stage accumulation consumes N×(11+H) clock periods using the traditional accumulation scheme. If the two kinds of image sensors work in the same conversion rate, the ratio of the line rate between them could be expressed as

(2) $$\mathrm{line}\;\mathrm{rate}\;\mathrm{ratio}=\frac{N\times (11+H)}{\left(N+4)\times (11-4+H\right)}.$$

Since the power consumption is proportional to the line rate, the low power accumulation scheme could achieve lower power consumption than the traditional accumulation scheme with the same line rate. The saved power consumption increases with the increase of accumulation stages for the fixed coarse quantization bit numbers and coarse quantization times.

### 2.3. MATLAB Behavioral Simulation

**Figure 5:**
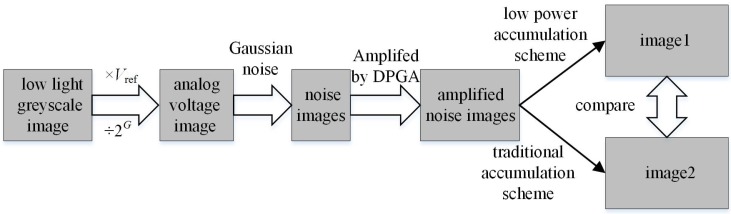
Block diagram of the MATLAB behavioral simulation.

**Figure 6:**
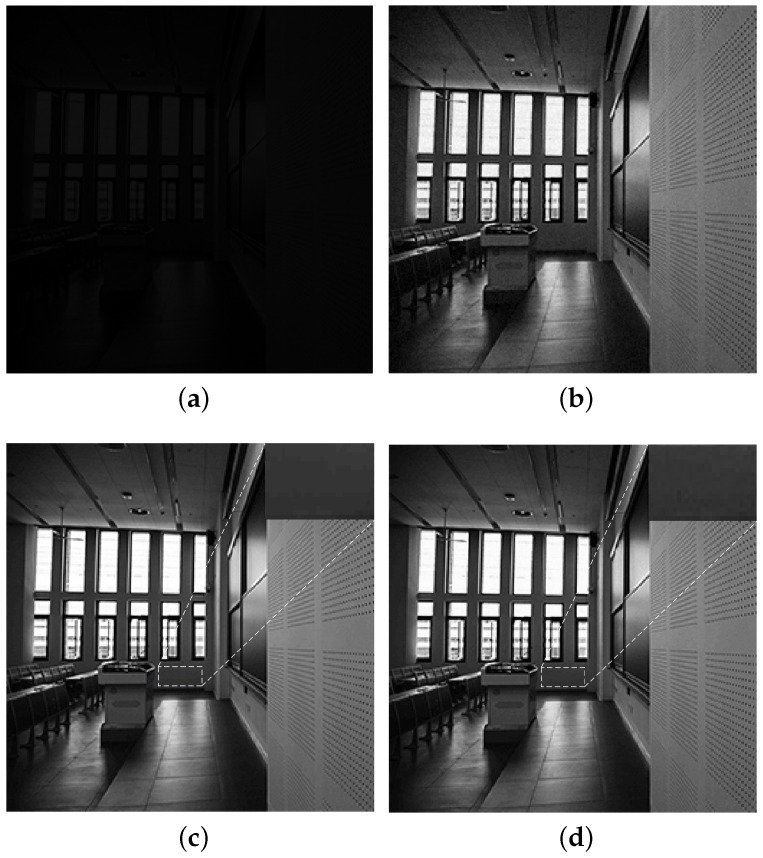
Greyscale images (**a**) Greyscale image with auxiliary noise; (**b**) Amplified image; (**c**) Reconstructed image processed by the traditional accumulation scheme; and (**d**) Reconstructed image processed by the low power accumulation scheme.

In order to verify the efficiency of the low power accumulation technique, a behavioral simulation is completed with MATLAB to compare the imaging quality between the proposed and the traditional 16-stage digital domain accumulation schemes. The brief block diagram of the behavioral model is shown in Figure 5. First, a 256×256 greyscale image is obtained under the low illumination condition, and then the greyscale image is transferred to analog voltage array according to the performance of SAR ADC. The terms ’$V_{ref}$’ and ’*G*’ shown in Figure 5 indicate the quantization range and precision of SAR ADC, respectively. According to the voltage information, the Gaussian noise is mapped to the greyscale image. Then, the greyscale images with Gaussian noise are amplified by digital programmable gain amplifier (DPGA) and are handled with the two kinds of accumulation schemes. The greyscale image with auxiliary Gaussian noise is shown in Figure 6a and the image that is amplified by DPGA is shown in Figure 6b. Figure 6c,d illustrate the reconstructed images processed with the two kinds of accumulation schemes. There are no more differences between the two images from the visual effect.

## 3. Circuit Implementation and Simulation Results

### 3.1. Circuit Implementation

**Figure 7:**
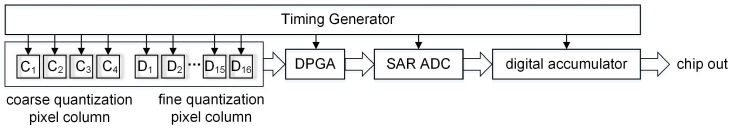
Block diagram of the 16-stage low power digital domain CMOS TDI image sensor chain.

In this work, two versions of 16-stage digital domain CMOS TDI image sensor chains, with and without the low power accumulation technique, are designed in the 0.18 µm CMOS process. The image sensor chain consists of one column pixel and one column readout circuit. According to the analysis in Section 2, 4-bit coarse quantization and four lines of pixels for coarse quantization are selected in the 16-stage low power version. The low power digital domain CMOS TDI image sensor chain is shown in Figure 7. It consists of a pixel column, a DPGA, a 10-bit SAR ADC and a digital accumulator. The pixel column consists of a coarse quantization pixel column and a fine quantization pixel column.

#### 3.1.1. Pixel and DPGA

**Figure 8:**
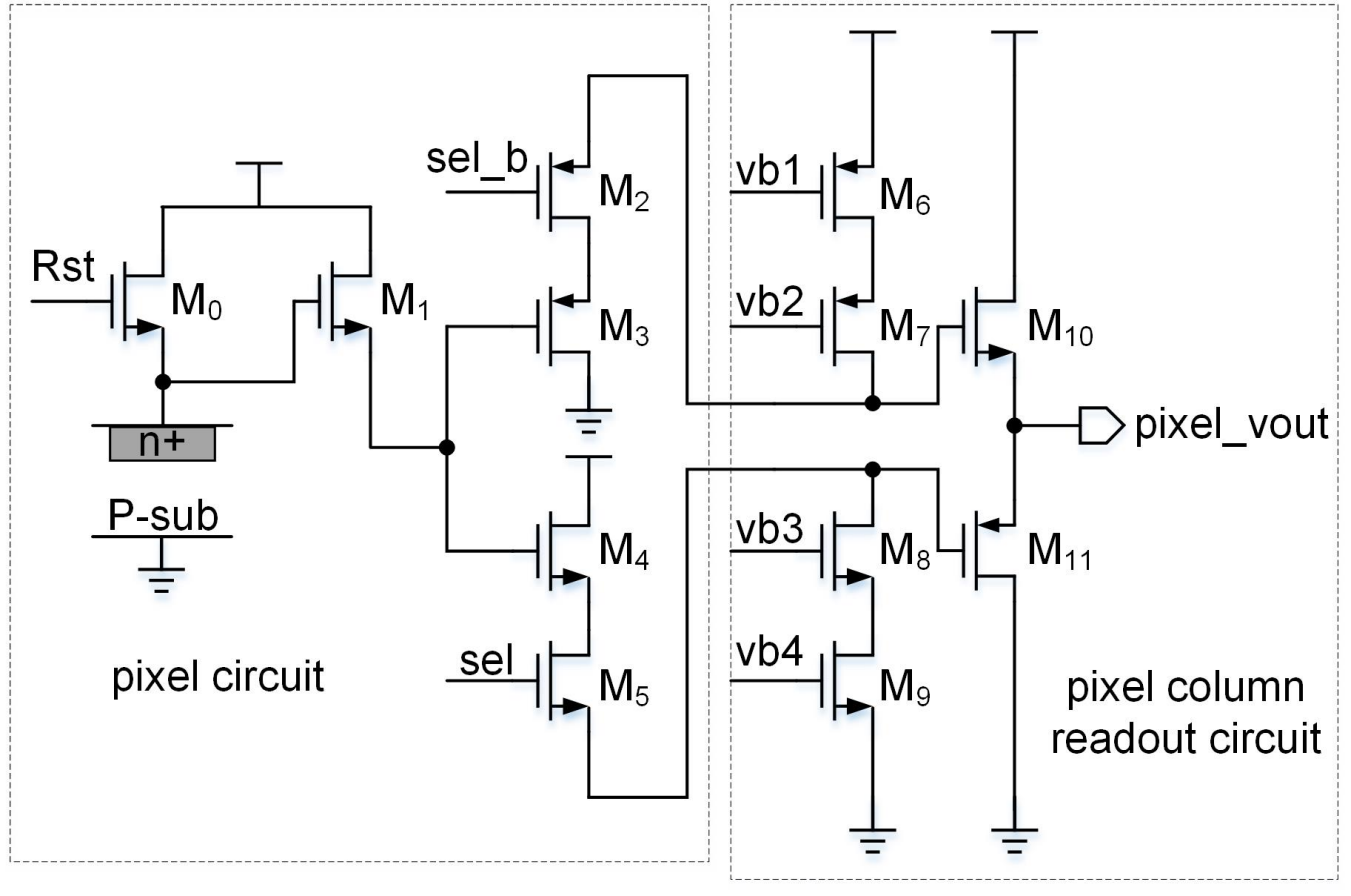
Schematic of the pixel circuit.

**Figure 9:**
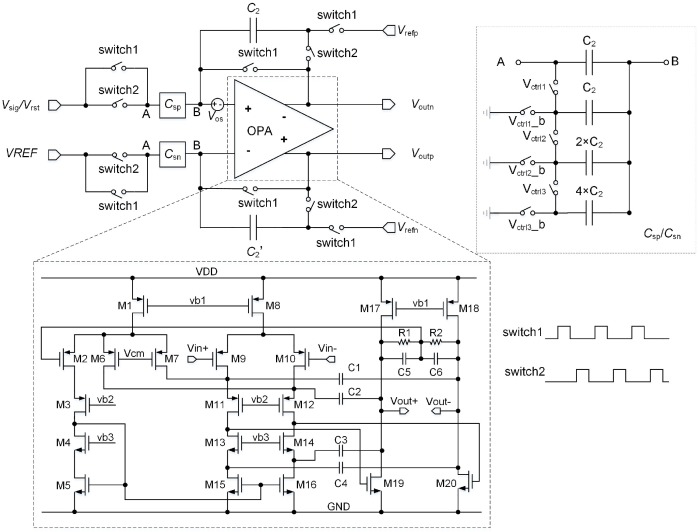
Schematic of the DPGA.

As is shown in Figure 8, the pixel circuit consists of a normal 3T structure and a class AB output stage for driving the large load capacitor provided by the DPGA. The schematic of the DPGA is shown in Figure 9. It is composed of some switches, one group capacitor of $C_{2}=C_{2}^{\prime }$, one group programmable capacitor array of $C_{\mathrm{sp}}=C_{\mathrm{sn}},$ and a two-stage fully differential operational amplifier (OPA) [15] with hybrid cascade compensation technique [16]. The simulation results of the OPA are shown in Table 1 with a 3.3 V supply voltage and 5 pF capacitance load. The corners ‘ss’, ‘tt’ and ‘ff’ are defined as (NMOS: slow and PMOS: slow), (NMOS: typical and PMOS: typical) and (NMOS: fast and PMOS: fast).

#### 3.1.2. SAR ADC

**Figure 10:**
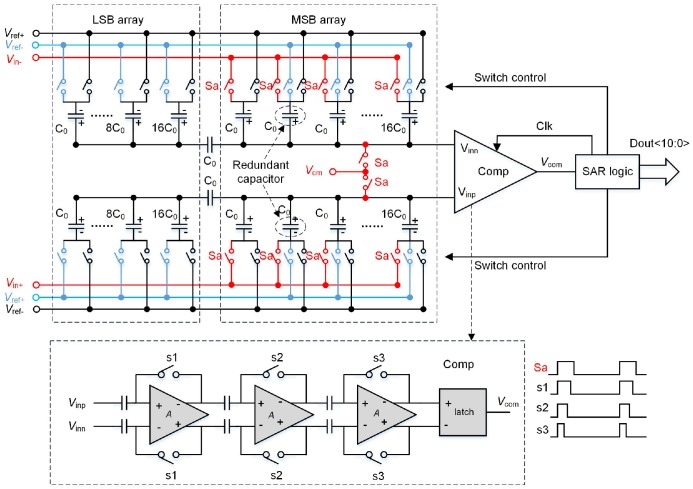
Schematic of the SAR ADC.

**Figure 11:**
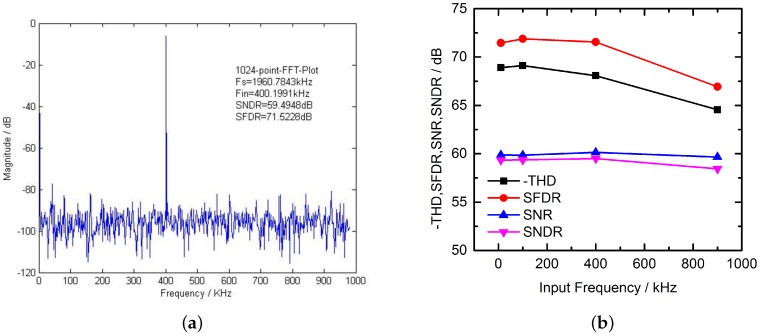
Simulation results of the SAR ADC (**a**) 1024-point FFT spectrum at 1.96 MS/s of SAR ADC; and (**b**) dynamic performance of SAR ADC versus input frequency.

The SAR ADC uses a split-capacitor structure with redundancy and a digital correction technique [17]. The schematic of the SAR ADC is shown in Figure 10. It is composed of a digital-to-analog converter (DAC), a comparator and one SAR logic. The comparator of the SAR ADC uses three cascaded low-gain amplifiers as the preamplifier and a latch, applying a dynamic input offset cancellation technique [18]. Thus, the input offset is quite small [19]. Figure 11a shows the simulation results of the fast Fourier transform (FFT) spectrum of the SAR ADC with an input frequency close to 400 KHz at a 1.96 MS/s sampling rate. Figure 11b plots the simulation results of total harmonic distortion (THD), spurious free dynamic range (SFDR), SNR, signal to noise distortion ratio (SNDR) versus the input frequency at 1.96 MS/s sampling rate.

#### 3.1.3. Digital Accumulator

**Figure 12:**
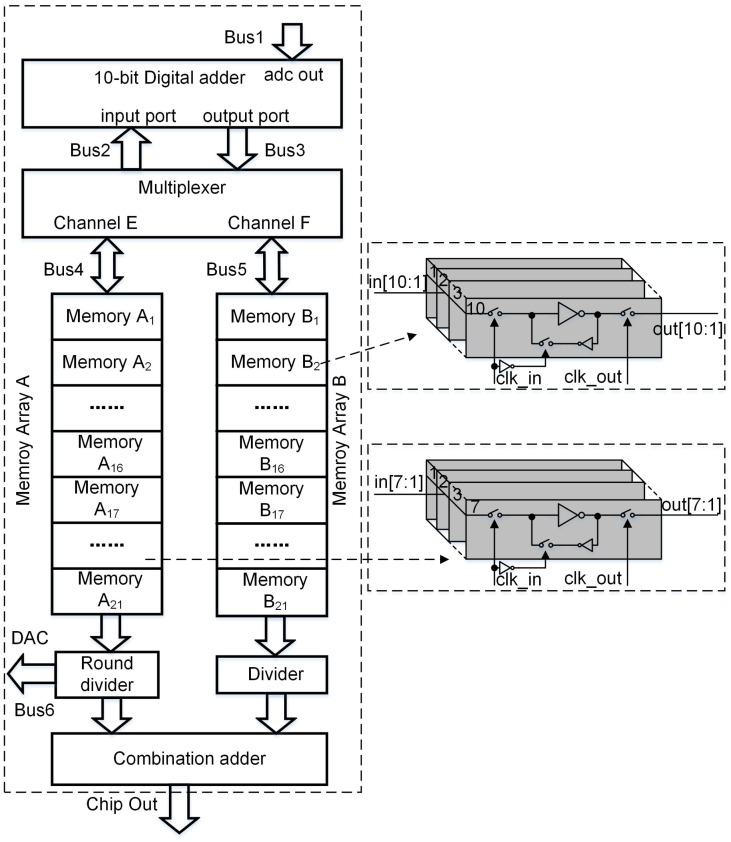
Block diagram of the digital accumulator.

The block diagram of the digital accumulator is shown in Figure 12. It consists of a 10-bit digital adder, a multiplexer, a memory array A, a memory array B, a combination adder, a divider, a round divider, three one-way buses (bus1 to bus3), and three two-way buses (bus4 to bus6). The memory array A is used to store the coarse quantization results, and the memory array B is used to store the fine quantization results. The combination adder is used to combine separate quantization results, and the round divider is used to calculate the round average value of the accumulated high-bit codes. As the discussion above, the coarse quantization pixel column consists of four lines ($C_{1}$–$C_{4}$) of pixels and the fine quantization pixel column is composed of 16 lines ($D_{1}$–$D_{16}$) of pixels. Based on the oversampling exposure method [20], 2×(4+6+1) memories are needed to store the results of coarse quantization and fine quantization.

The detailed operation procedure of the digital accumulator is described as follows. Because the SAR ADC uses a redundancy method, the actual bit numbers of coarse quantization increase to 5 bits. When the image sensor starts to work, the four lines coarse quantization pixels ($C_{1}$–$C_{4}$) perform in advance to capture the object four times. The output result of the $C_{1\mathrm{st}}$ line of pixels undergoes the coarse quantization by SAR ADC, and then the quantized high 5-bit data are stored in the memory $A_{16}$. After that, the output result from the $C_{2\mathrm{nd}}$ line of pixels also completes the coarse quantization through SAR ADC, and the quantized digital codes are transferred to the digital adder via bus1. The data stored in the memory $A_{16}$ is also delivered to the digital adder through bus4, multiplexer and bus2. Then, the output of the digital adder is sent back to the memory $A_{16}$ through bus3, multiplexer and bus4. With the same technique, the signals output by four lines of coarse quantization pixels are quantized and accumulated. Then, the accumulated result is stored in the memory $A_{16}$.

During the fine quantization, after the $D_{1\mathrm{st}}$ line of pixels finishes the exposure of the same object, the result is sampled by SAR ADC. In the meantime, the round average value of the accumulated data stored in the memory $A_{16}$ is sent back to the DAC of SAR ADC through bus6. Based on the coarse quantization results, the SAR ADC performs the remaining successive approximation to the analog input signal, which is defined as the fine quantization. The quantized digital codes are stored in the memory $B_{16}$. In a similar manner, the signal stored in the memory $B_{16}$ is accumulated 16 times. Then, the round average value of the data stored in the memory $A_{16}$ is delivered to an input port of the combination adder. Meanwhile, the data stored in the memory $B_{16}$ is divided by 16, and the quotient is also delivered to the other port of the combination adder. Finally, the output of the combination adder is served as the complete quantization result of the scanned object.

#### 3.1.4. Noise Analysis of the Low Power Readout Circuit

The noise of the low power digital domain readout circuit is mainly contributed by the pixel circuit, the DPGA and the SAR ADC. There are two important intrinsic noise sources: thermal noise and flicker noise [21]. The flicker noise of the pixel circuit and DPGA are reduced to a negligible level by using theinut input–offset storing technique [22]. Because the pixel uses a 3T structure, *KTC* noise could not be eliminated. Benefitting from the DPGA, the total equivalent input noise of DPGA and SAR ADC is divided by the amplified magnitude of the DPGA. Therefore, we mainly concentrate on the effect of the thermal noise contributed by the source follower. Assuming that the source follower is equivalent to a unity-gain amplifier and the total equivalent output power spectral density of the noise of the source follower could be expressed as

(3) $$\begin{array}{c} V_{\mathrm{nSF}\_\mathrm{out}}{\left(f\right)}^{2}=4kT\gamma \times \left(\frac{1}{g_{m1}}+\frac{1}{g_{m3}}+\frac{1}{g_{m4}}+\frac{1}{g_{m10}}+\frac{1}{g_{m11}}\right)\\ \;\;\;\;\;\;\;\;\;\;\;\;\;\;\;\;\;\;\;\;\;\;\;\;\;\;\;\;\;\;\;\;\;\;\;\;\;\;\;\;\;\;\;\;\;\;\;\;+\frac{4kT\gamma }{g_{m3}^{2}}\times \left(g_{m2}+g_{m6}+g_{m7}\right)\;+\frac{4kT\gamma }{g_{m4}^{2}}\times \left(g_{m5}+g_{m8}+g_{m9}\right), \end{array}$$

where *k* is the Boltzmann constant, *T* is the absolute temperature, and *γ* is a coefficient of process. Since the source follower could be equivalent to a single-pole system, the equivalent bandwidth of noise could be expressed as

(4) $$BW_{nSF\_equ}=\frac{1}{2}\times \frac{g_{m1}}{2\times (C_{\mathrm{bus}}+C_{s})},$$

where $C_{\mathrm{bus}}$ and $C_{s}$ are the total node parasitic capacitance and load capacitance of the pixel readout circuit. Thus, the total equivalent output noise of the source follower could be expressed as

(5) $$\begin{array}{c} \bar{{V_{\mathrm{nSF}\_\mathrm{out}}}^{2}}=\int_{1}^{BW_{\mathrm{nSF}\_\mathrm{equ}}}V_{\mathrm{nSF}\_\mathrm{out}}{\left(f\right)}^{2}df=\frac{g_{m1}\times \xi_{1}}{\left(C_{\mathrm{bus}}+C_{s}\right)}\\ \xi_{1}=kT\gamma \times \left(\frac{1}{g_{m1}}+\frac{1}{g_{m3}}+\frac{1}{g_{m4}}+\frac{1}{g_{m10}}+\frac{1}{g_{m11}}+\frac{g_{m2}}{g_{m3}^{2}}+\frac{g_{m6}}{g_{m3}^{2}}+\frac{g_{m7}}{g_{m3}^{2}}+\frac{g_{m5}}{g_{m4}^{2}}+\frac{g_{m8}}{g_{m4}^{2}}+\frac{g_{m9}}{g_{m4}^{2}}\right). \end{array}$$

The noise optimization of the source follower is completed by properly increasing $g_{m3-4}$, $g_{m10-11}$ and reducing $g_{m2}$, $g_{m5-9}$. Since the output signals of pixels are amplified *H* times, the total equivalent input noise contributed by the readout circuit after *N* times accumulation could be expressed as

(6) $$\bar{V_{\mathrm{Ntot}}}=\sqrt{N\times \left(\frac{kT}{C_{\mathrm{PD}}}+\bar{{V_{\mathrm{nSF}\_\mathrm{out}}}^{2}}\right)+\frac{N}{H^{2}}\times \left(\bar{{V_{\mathrm{nDPGA}\_\mathrm{out}}}^{2}}+\bar{{V_{\mathrm{nADC}\_\mathrm{out}}}^{2}}\right)},$$

where $C_{\mathrm{PD}}$ is the photo-diode node capacitance, $\bar{{V_{\mathrm{nDPGA}\_\mathrm{out}}}^{2}}$ is the noise energy contributed by the DPGA, and $\bar{{V_{\mathrm{nADC}\_\mathrm{out}}}^{2}}$ is the noise energy contributed by the SAR ADC.

### 3.2. Linearity Simulation Results and Power Consumption Analysis

**Figure 13:**
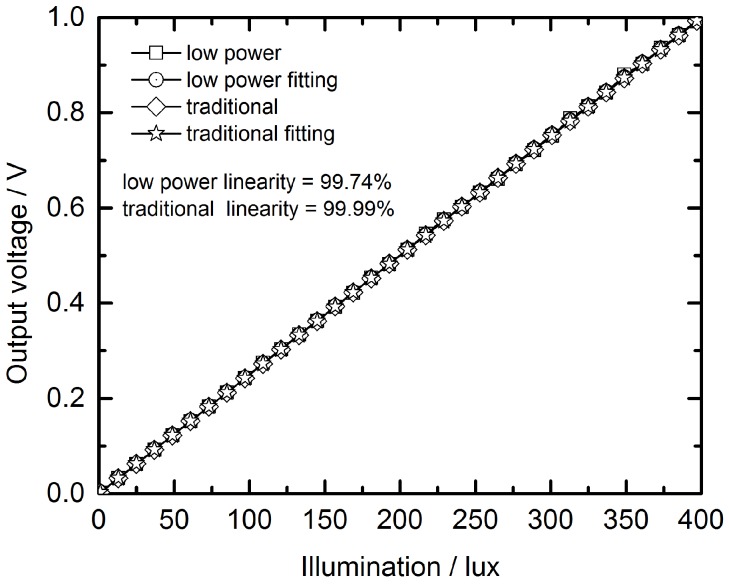
Linearity simulation results of the two versions.

According to the non-linearity measured method of CMOS image sensors [23], the linearity is defined as

(7) $$\mathrm{Linearity}=(1-\frac{\Delta V_{\max}}{V_{\max}})\times 100\%,$$

where the $\Delta V_{\max}$ is the maximum difference between the simulated and fitted transmission curves and the $V_{\max}$ is the full scale. The linearity simulation results of the two versions are shown in Figure 13. The linearity of the image sensor chains are 99.74% with the low power accumulation scheme and 99.99% with the traditional accumulation scheme.

**Figure 14:**
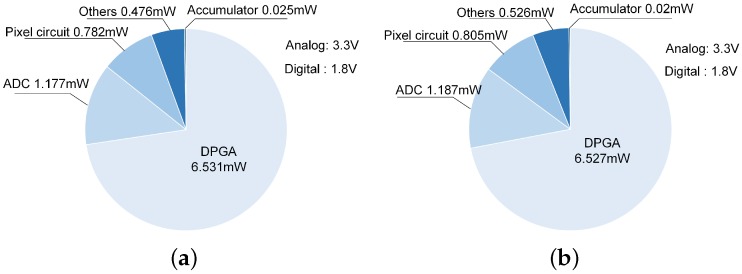
Power consumption distributions of the two kinds of image sensor chains (**a**) low power accumulation scheme; and (**b**) traditional accumulation scheme.

The total power consumption of the image sensor chain is contributed by the DPGA, the SAR ADC, the accumulator, the pixel circuit and other circuits. The other circuits include a brief current generation circuit and some digital buffers. The distributions of the power consumptions of the two versions are shown in Figure 14. The comparisons of some main parameters of the two kinds of image sensor chains with our prior works are shown in Table 2. Compared with our prior works and the traditional accumulation scheme, the proposed low power digital accumulation technique can achieve lower average power consumption of each slice. The average power consumption of each slice is defined as

(8) $$\mathrm{average}\;\mathrm{power}\;\mathrm{consumption}\;\mathrm{of}\;\mathrm{slice}=\frac{\mathrm{total}\;\mathrm{power}\;\mathrm{consumption}}{\left(\mathrm{horizontal}\;\mathrm{resolution})\times (\mathrm{line}\;\mathrm{rate}\right)}.$$

## 4. Conclusions

In this paper, a low power digital accumulation technique for a digital domain CMOS TDI image sensor is proposed to reduce power consumption without degrading the rate of imaging. The low power accumulation scheme is composed of coarse quantization and fine quantization. The optimized bit numbers and times of coarse quantization are analyzed. With the 0.18 µm CMOS process, two versions of 16-stage digital domain CMOS TDI image sensor chains, with and without the proposed technique, were designed. The simulation results show that the average power consumption of each slice are $6.47\times 10^{-8}$ J/line and $7.4\times 10^{-8}$ J/line, respectively. The linearity of the image sensor chains are 99.74% with the low power accumulation scheme and 99.99% with the traditional accumulation scheme.

## Figures and Tables

**Table 1:** Simulation results of the OPA.

Corner	Temperature	Supply Voltage of OPA	Output Voltage Swing of OPA	Unity Gain-Bandwidth	Gain	Phase Margin
tt	−40${}^{\circ }$C–80${}^{\circ }$C	2.97 V –3.63 V	0.85 V–2.45 V	135 MHz–167 MHz	105 dB–117 dB	68${}^{\circ }$–69${}^{\circ }$
ss	−40${}^{\circ }$C–80${}^{\circ }$C	2.97 V –3.63 V	0.85 V–2.45 V	118 MHz–161 MHz	107 dB–117 dB	67${}^{\circ }$–69${}^{\circ }$
ff	−40${}^{\circ }$C–80${}^{\circ }$C	2.97 V–3.63 V	0.85 V–2.45 V	124 MHz–173 MHz	97 dB–116 dB	68${}^{\circ }$–71${}^{\circ }$

**Table 2:** Comparisons of some main parameters of the two kinds of image sensor chains with prior works. (H-Horizontal, V-Vertical).

Parameter	This Work		[10]	[14]
	Low Power	Traditional		
Technology	0.18 µm CMOS	0.18 µm CMOS	0.18 µm CMOS	0.18 µm CMOS
Array size	1 (H)×20 (V)	1 (H) ×16 (V)	1024 (H)×128 (V)	1024(H)×128 (V)
Maximum stage	16	16	128	128
Maximum line rate	138888 lines/s	122549 lines/s	3875 lines/s	3875 lines/s
Total power consumption	8.991 mW	9.065 mW	500 mW	290 mW
Average power consumption of slice	$6.47\times 10^{-8}$ J/line	$7.4\times 10^{-8}$ J/line	$12.6\times 10^{-8}$ J/line	$7.3\times 10^{-8}$ J/line
